# Bilateral posterior tibial nerve stimulation as a neuromodulation strategy for obstructed defecation: a randomized controlled trial

**DOI:** 10.1007/s10151-025-03231-2

**Published:** 2025-10-16

**Authors:** Anwar Ashraf Abouelnasr, Mohamed Hany

**Affiliations:** 1 Medical Research Institute, Alexandria University, Alexandria, Egypt; 2 Madina Women‘s Hospital, Alexandria, Egypt

**Keywords:** Obstructed defecation syndrome, Posterior tibial nerve stimulation, Electromyography, Pelvic floor disorders, Noninvasive therapy

## Abstract

**Background:**

Obstructed defecation syndrome (ODS) is a prevalent pelvic floor disorder, often impairing patients’ quality of life. Noninvasive therapies, including posterior tibial nerve stimulation (PTNS), have been explored as alternative treatments. This study evaluates the efficacy of bilateral transcutaneous posterior tibial nerve stimulation (BT-PTNS) compared to medical treatment alone in patients with ODS without anatomical abnormalities.

**Methods:**

A prospective randomized controlled study was conducted on 50 patients diagnosed with ODS. Patients were randomly assigned into two groups: group A received BT-PTNS sessions three times weekly for 6–12 weeks alongside medical treatment, while group B received medical treatment only. Outcomes were assessed using the Modified Obstructed Defecation Syndrome (MODS) score, Patient Assessment of Constipation Quality of Life (PAC-QOL) questionnaire, and quantitative electromyography of pelvic floor muscles. Statistical analysis was performed using SPSS software.

**Results:**

Group A exhibited a significant reduction in MODS scores (mean decrease = 10 points) compared to group B (mean decrease = 4 points) after 6 weeks (*p* < 0.001). PAC-QOL scores improved significantly in group A (65% reduction) compared to group B (37% reduction). Electromyographic analysis in group A showed significant improvement in amplitude, number of motor unit turns, and duration (*p* < 0.001). No adverse events were reported in either group.

**Conclusion:**

BT-PTNS is a safe and effective noninvasive treatment for ODS without anatomical abnormalities, significantly improving symptom severity and quality of life. Further multicentric trials are warranted to standardize treatment protocols and assess long-term outcomes.

**Trial registration:**

Clinical Trial Number IORG0008812; E/C.S/N.R2/2017

## Introduction

Obstructed defecation syndrome (ODS) is a subtype of functional constipation characterized by impaired rectal evacuation despite normal or increased rectal sensation. It significantly impacts the quality of life, particularly among women and older adults, and poses a substantial burden on healthcare systems [1–5]. While anatomical abnormalities such as rectocele or intussusception are known contributors, many patients present without structural defects and are diagnosed with functional ODS [6–9].

According to the Rome III criteria, functional constipation is defined as at least two symptoms, such as straining, lumpy or hard stools, sensation of incomplete evacuation, anorectal blockage, or manual maneuvers to facilitate defecation, occurring for at least 3 months [10]. ODS specifically refers to a subset of these patients with outlet dysfunction, often involving paradoxical puborectalis contraction or impaired pelvic floor coordination [11].

Conservative management of functional ODS typically includes dietary fiber supplementation, laxatives, behavioral therapy, and pelvic floor rehabilitation [12–14]. While these methods are often the first line of treatment, their effectiveness can be limited, particularly for patients with pelvic floor dyssynergia, which is a condition where the pelvic floor muscles do not relax properly during defecation [15, 16]. In this context, neuromodulation therapies have emerged as promising noninvasive alternatives.

Posterior tibial nerve stimulation (PTNS) involves electrical stimulation of a peripheral nerve that shares sacral roots (L4–S3) with the pelvic floor, bladder, and lower bowel innervation [17]. Stimulating the posterior tibial nerve may enhance stool evacuation through the activation of S3 and/or S2 pathways [17]. Its clinical utility has been demonstrated in managing overactive bladder, fecal incontinence, and other pelvic floor dysfunctions [17–20]. The underlying mechanism is presumed to involve modulation of somatic and autonomic efferent pathways.

Preliminary data suggest PTNS may also benefit patients with functional constipation and ODS, with promising outcomes reported in small trials and observational studies [21–23]. Nonetheless, randomized evidence, especially using bilateral transcutaneous stimulation, remains limited.

This randomized controlled trial aimed to evaluate the efficacy of bilateral transcutaneous posterior tibial nerve stimulation (BT-PTNS) compared to standard medical therapy in patients with functional ODS, using validated symptom severity and quality-of-life assessment tools.

## Methods

This prospective, randomized controlled, parallel-group trial was conducted at the Colorectal Surgery Unit, Faculty of Medicine, Alexandria University, between August 2017 and July 2018. The study was approved by the local institutional ethics committee (IORG0008812; E/C.S/N.R2/2017) and conducted following the principles of the Declaration of Helsinki. All participants provided written informed consent before enrollment.

Eligible participants were adults diagnosed with ODS of functional origin, defined as the presence of at least two core symptoms in more than 25% of defecation attempts, including straining, hard or lumpy stools, urgency, a sensation of incomplete evacuation, pelvic heaviness, or the need for manual assistance. To exclude anatomical abnormalities, all patients underwent either high-resolution dynamic pelvic magnetic resonance imaging or fluoroscopic defecography. Patients with structural abnormalities such as rectocele, enterocele, rectal intussusception, or patients with a history of anal fissures were excluded. Additional exclusion criteria included congenital anorectal anomalies, diabetes mellitus, hypothyroidism, known neurological disorders, epilepsy, pregnancy, and the presence of electronic implants such as pacemakers.

Participants were randomized in a 1:1 ratio to one of two treatment groups using a computer-generated random sequence. Allocation concealment was achieved using sealed, opaque envelopes, prepared and maintained by an independent researcher not involved in patient recruitment or outcome assessment.

**Fig. 1:**
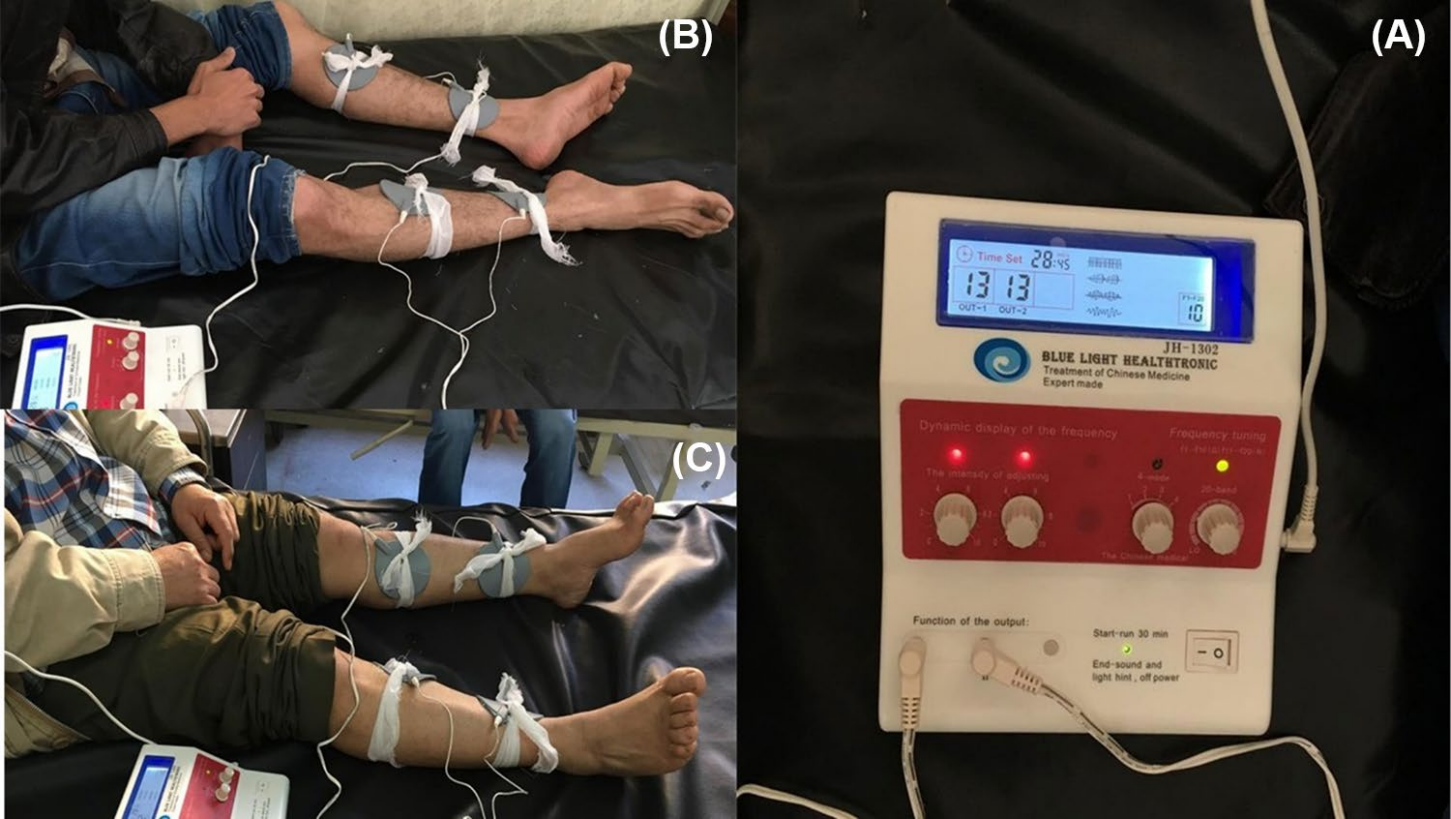
Electrode placement and stimulation settings for bilateral transcutaneous posterior tibial nerve stimulation (BT-PTNS). **a** TENS device settings; **b**, **c** electrode positioning with the cathode placed posterior to the medial malleolus and the anode 10 cm superiorly, bilaterally

Patients in the intervention group received BT-PTNS in addition to standard medical therapy. Electrical stimulation was delivered using a NeuroTrack transcutaneous electrical nerve stimulation (TENS) device, applied three times per week for a minimum of 6 weeks and up to 12 weeks, depending on the patient’s rate of improvement (Fig. 1). Adhesive electrodes were positioned with the negative electrode (cathode) placed posterior to the medial malleolus and the positive electrode (anode) positioned approximately 10 cm superior to it on the same leg. Stimulation parameters were standardized to a pulse width of 200 μs and a frequency of 10 Hz. The expected response included plantar flexion of the toes on the same side, without any sensation or contraction occurring in the pelvic area. All patients in this group also received standard medical management, including dietary fiber supplementation, adequate fluid intake, and osmotic laxatives such as polyethylene glycol or lactulose, adjusted according to individual tolerance.

The control group received the same medical therapy without neuromodulation. Both groups were treated and followed over a comparable 6–12-week period, depending on clinical response.

The primary outcome was the change in the Modified Obstructed Defecation Syndrome (MODS) score measured before treatment (baseline) and again at 6 weeks post treatment. Secondary outcomes included changes in the Patient Assessment of Constipation–Quality of Life (PAC-QoL) scores and quantitative electromyography (EMG) parameters of pelvic floor muscle function, both assessed at baseline and after 6 weeks. EMG parameters included signal amplitude (in millivolts), number of motor unit turns, and motor unit duration (in milliseconds), recorded during voluntary contraction. MODS and PAC-QoL are validated assessment tools previously described in the literature and are not elaborated upon in this report to maintain focus and conciseness [24, 25].

Sample size was calculated using a power analysis based on an anticipated effect size of 0.8 across the three main outcome measures. To achieve 80% statistical power at a significance level of 5%, 25 patients per group were required. Allowing for potential attrition, a total of 50 patients were enrolled.

Statistical analysis was performed using IBM SPSS Statistics version 20.0 (IBM Corp., Armonk, NY, USA). The normality of distribution was assessed using the Kolmogorov–Smirnov test. For categorical variables, comparisons between groups were conducted using the chi-square test or Fisher’s exact correction when appropriate. Non-normally distributed quantitative variables were compared using the Mann–Whitney* U* test for between-group comparisons and the Wilcoxon signed-rank test for within-group comparisons. Paired *t* tests were used for normally distributed EMG data. A* p* value less than 0.05 was considered statistically significant for all comparisons.

## Results

**Fig. 2:**
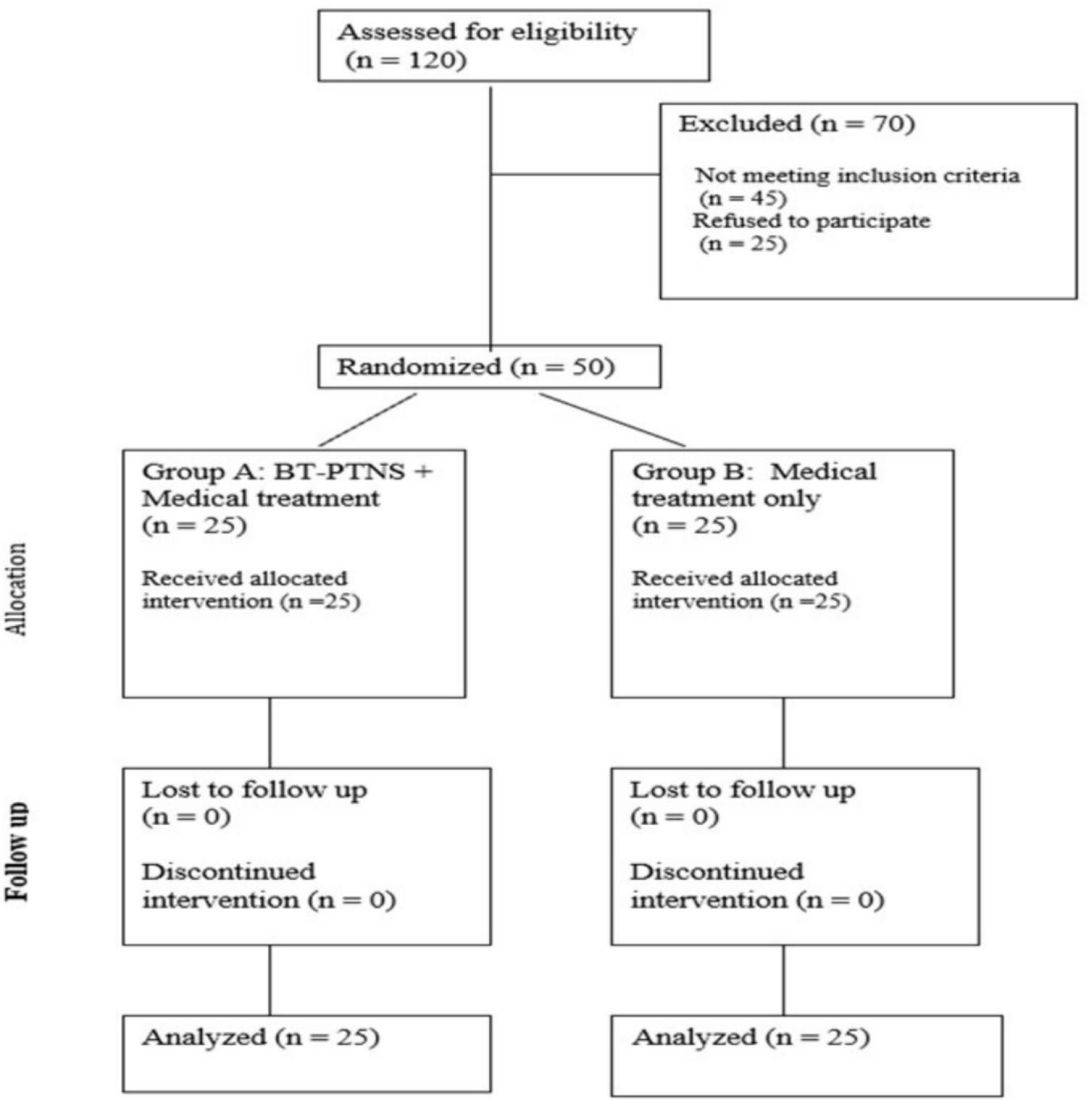
Flow diagram of participant enrollment, allocation, and follow-up according to CONSORT. A total of 120 patients were screened; 70 were excluded, and 50 were randomized equally between group A (BT-PTNS + medical therapy) and group B (medical therapy alone). All participants completed the study

A total of 120 patients were assessed for eligibility between August 2017 and July 2018. Seventy patients were excluded: 45 did not meet the inclusion criteria, and 25 declined to participate. Fifty patients were enrolled and randomized equally into two groups: 25 patients received bilateral posterior tibial nerve stimulation (group A), and 25 received standard medical therapy alone (group B). All participants completed the full course of treatment and follow-up (Fig. 2).

**Fig. 3:**
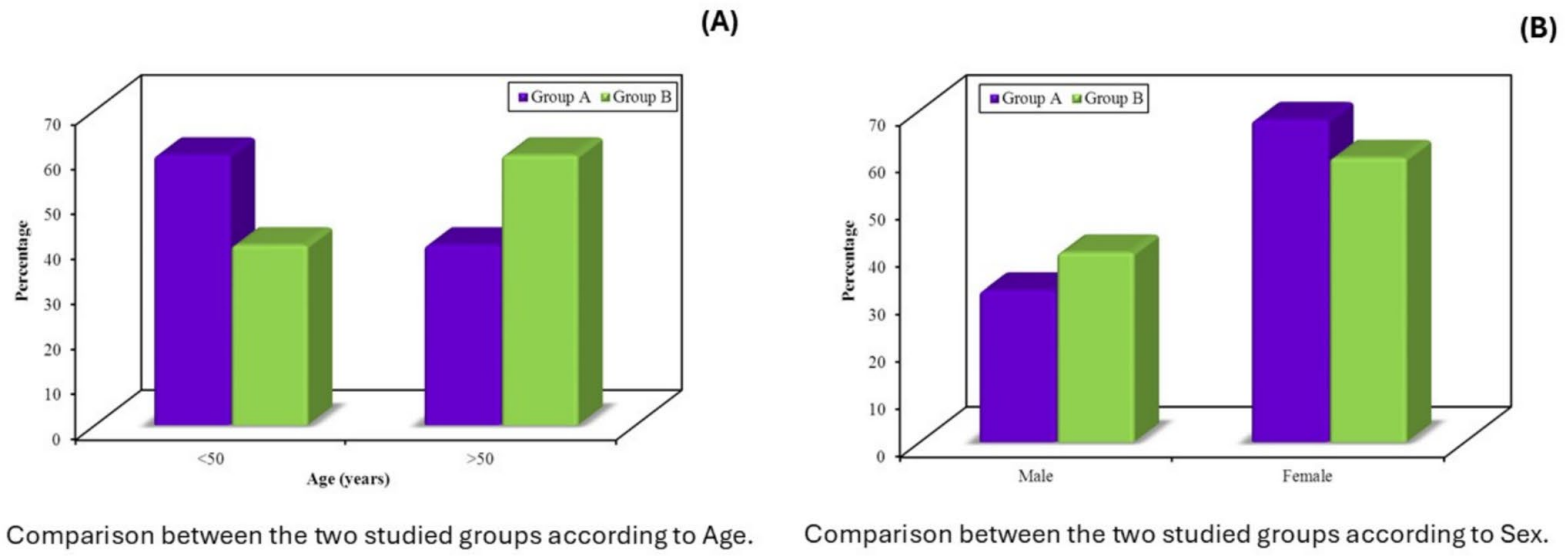
Baseline group comparison by demographics. **a** Age distribution; **b** sex distribution

**Fig. 4:**
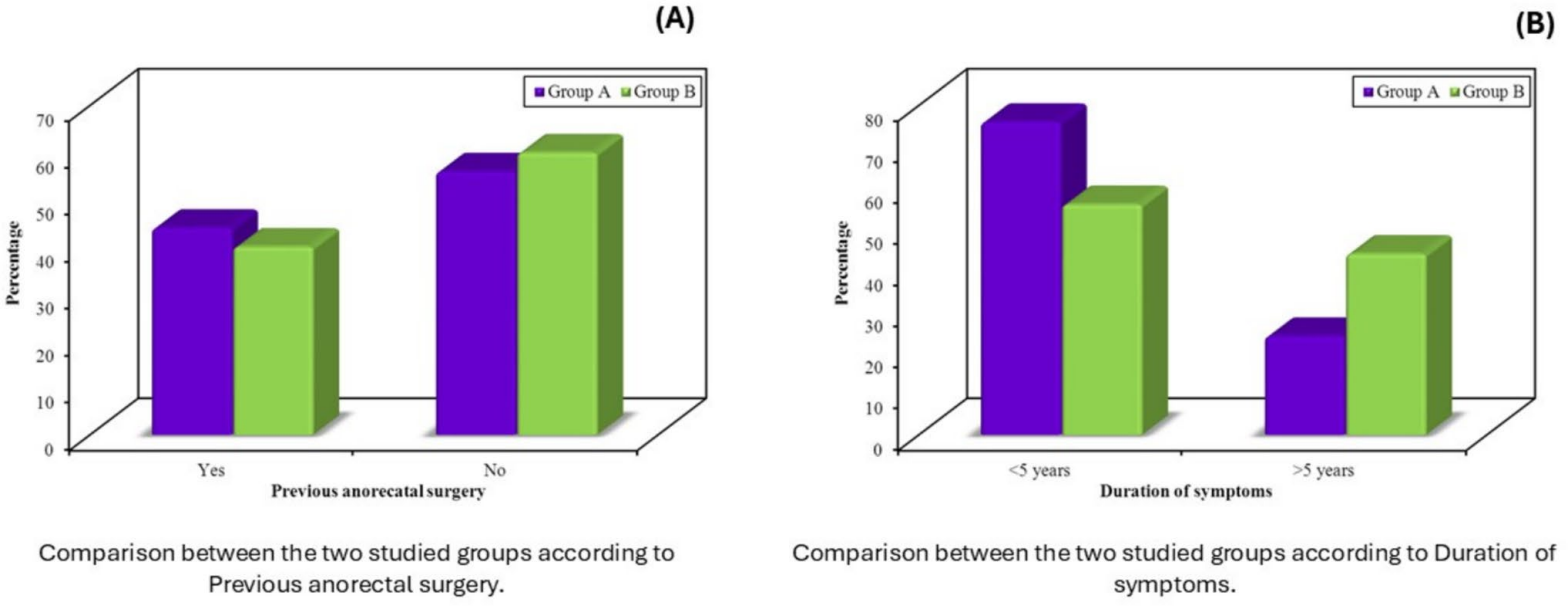
Baseline group comparison clinical features. **a** Prior anorectal surgery; **b** duration of symptoms

Baseline demographic and clinical characteristics were comparable between the two groups. Most participants were aged between 30 and 60 years. The sex distribution was similar, with group A comprising 17 women and 8 men, and group B comprising 15 women and 10 men (Table 1, Fig. 3). Prior anorectal surgery was reported in 11 women (64.7%) in group A and 10 men (100%) in group B, with statistically significant differences observed both within and across groups (*p* < 0.001) (Table 2, Fig. 4). Age distribution also differed significantly by sex: all men in both groups were older than 50 years, whereas the majority of women were younger than 50 (group A: *p* < 0.001; group B: *p* = 0.001) (Table 2). Duration of symptoms also varied, with longer symptom history more frequently reported in male participants in both groups (*p* < 0.001) (Table 2).

**Table 1:** Baseline demographic characteristics of study participants in group A (BT-PTNS) and group B (medical therapy)

	Group A (*n* = 25)		Group B (*n* = 25)		*χ*^2^	*p*
	No	%	No	%		
Age (years)						
< 50	15	60.0	10	40.0	2.000	0.157
> 50	10	40.0	15	60.0		
Sex						
Male	8	32.0	10	40.0	0.347	0.556
Female	17	68.0	15	60.0		

The two groups were comparable at baseline in terms of age and sex distribution. Data are presented as frequencies and percentages. Group A: Bilateral posterior tibial nerve stimulation. Group B: Control taking only medical treatment

*χ*^*2*^ chi-square test,* p* *p* value for comparison between the two groups

**Table 2:** Subgroup analysis by sex and treatment group, including symptom duration, prior anorectal surgery, and post-treatment outcomes

	Group A	Group B	Test statistic	P1 value
	Male (*n* = 8) Female (*n* = 17)	Male (*n* = 10) Female (*n* = 15)		
Age				
Male				
> 50 years	8 (100)	10 (100)	–	–
Female				
< 50 years	15 (88.2)	10 (66.7)	FE(χ^2^ = 1.971)	0.209
> 50 years	2 (11.8)	5 (33.3)		
P2 value	< 0.001*	0.001*		
Previous anorectal surgery				
Male	0 (0)	10 (100)	FE(χ^2^ = 18.00)	< 0.001*
Female	11 (64.7)	0 (0)	FE(χ^2^ = 14.79)	< 0.001*
P2 value	0.003*	< 0.001*		
Duration of symptoms				
Male			FE(χ^2^ = 2.813)	0.183
< 5 years	2 (25)	0 (0)		
> 5 years	6 (75)	10 (100)		
Female			FE(χ^2^ = 1.170)	0.469
< 5 years	17 (100)	14 (93.3)		
> 5 years	0 (0)	1 (6.7)		
P2 value	< 0.001*	< 0.001*		
MODS pre-treatment				
Male	17.63 ± 1.51	14.60 ± 1.65	*t* = 4.020	0.001*
Female	16.71 ± 1.57	16.33 ± 1.50	*t* = 0.684	0.499
P2 value	0.180	0.012*		
MODS after 6 weeks				
Male	12.38 ± 6.21	13.60 ± 1.51	*t* = − 0.606	0.553
Female	4.53 ± 0.72	10.20 ± 1.47	*t* = − 14.106	< 0.001*
P2 value	< 0.001*	< 0.001*		
PAC-QoL pre-treatment				
Male	51.13 ± 6.06	52.10 ± 6.03	*t* = − 0.340	0.738
Female	51.82 ± 5.70	51.60 ± 5.63	*t* = 0.111	0.912
P2 value	0.782	0.834		
PAC-QoL after 6 weeks				
Male	33.75 ± 21.00	49.80 ± 5.96	*t* = − 2.319	0.034*
Female	10.76 ± 2.36	20.67 ± 4.10	*t* = − 8.500	< 0.0018
P2 value	< 0.001*	< 0.001*		
EMG amplitude		–	–	**–**
Male				
Increased	3 (37.5)			
Decreased	5 (62.5)			
Female				
Increased	17 (100)			
P2 value	0.001*			
EMG turns and duration		–	–	–
Male				
Decreased	3 (37.5)			
Increased	5 (62.5)			
Female				
Decreased	17 (100)			
P2 value	0.001*			

Female participants, particularly in group A, demonstrated significantly greater improvement in MODS, PAC-QoL, and EMG parameters. Male participants in both groups were predominantly older and had longer symptom duration

*P1* *p* value for comparison of group A vs group B according to sex, *P2* *p* value for comparison of male vs female for each group

*Statistically significant at *p* ≤ 0.05

### Clinical outcomes

**Fig. 5:**
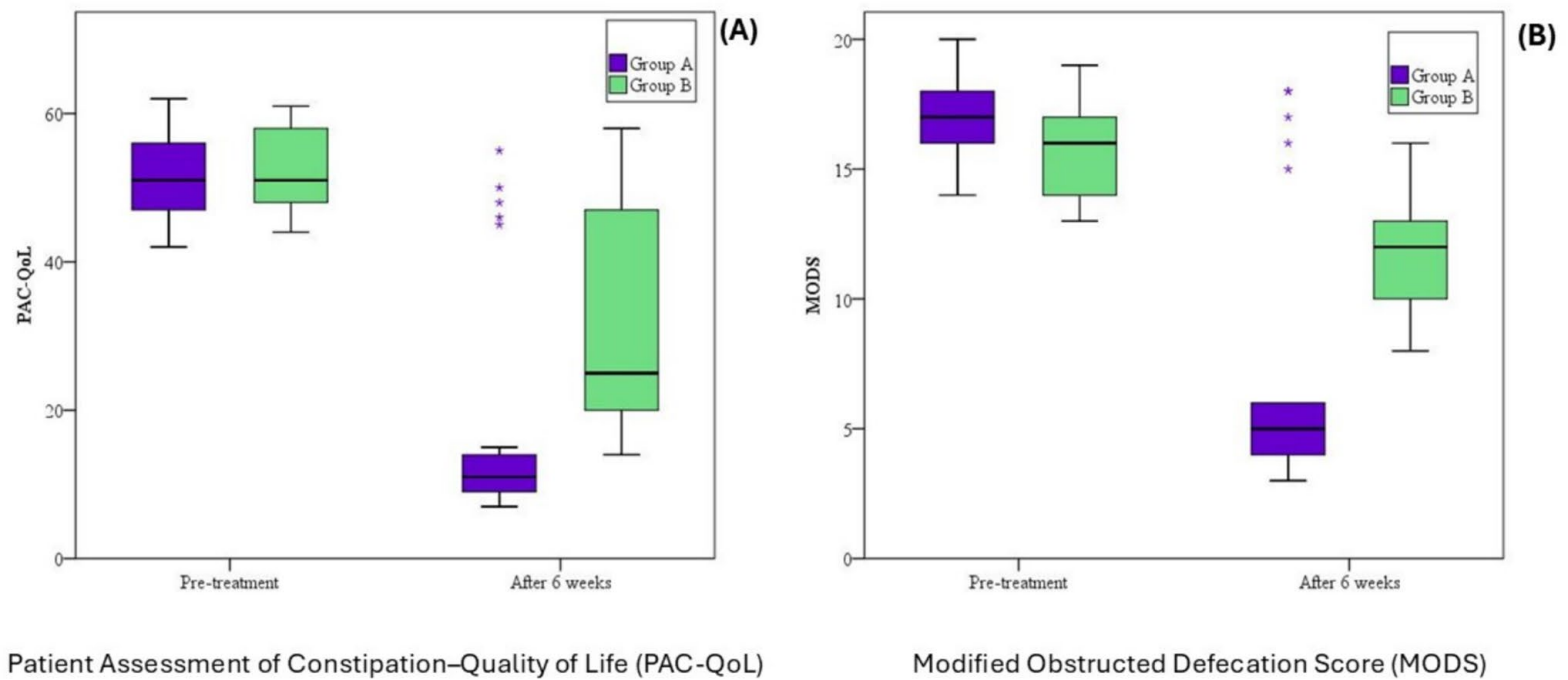
Comparison of clinical outcomes at baseline and after 6 weeks of treatment. **a** PAC-QoL scores; **b** MODS scores. Group A demonstrated significantly greater improvement than group B in both outcomes (*p* < 0.001)

Analysis of MODS values confirmed this improvement. In group A, MODS scores decreased from a mean of 17.0 ± 1.58 (median 17.0; range 14.0–20.0) at baseline to 7.04 ± 5.05 (median 5.0; range 3.0–18.0) at 6 weeks (*p* < 0.001). In group B, MODS also declined from 15.64 ± 1.75 (median 16.0; range 13.0–19.0) to 11.56 ± 2.24 (median 12.0; range 8.0–16.0) (*p* < 0.001). Between-group comparisons showed a significantly greater reduction in group A (*p* < 0.001) (Table 3, Fig. 5).

**Table 3:** Comparison of Modified Obstructed Defecation Syndrome (MODS) score before and after 6 weeks of treatment

MODS	Group A (*n* = 25)	Group B (*n* = 25)	*U*	*p*
Pre-treatment				
Min–Max	14.0–20.0	13.0–19.0	178.50*	0.008*
Mean ± SD	17.0 ± 1.58	15.64 ± 1.75		
Median	17.0	16.0		
After 6 weeks				
Min–Max	3.0–18.0	8.0–16.0	122.50*	< 0.001*
Mean ± SD	7.04 ± 5.05	11.56 ± 2.24		
Median	5.0	12.0		
p_1_	< 0.001*	< 0.001*		

Group A demonstrated a greater reduction in MODS scores compared to group B. Data are presented as mean ± SD. Within-group and between-group differences were statistically significant (*p *< 0.001). Group A: Bilateral posterior tibial nerve stimulation. Group B: Control taking only medical treatment

*U* Mann–Whitney test,* p* *p* value for comparison between the two groups,* P1 p* value for Wilcoxon signed ranks test for comparison between pre-treatment and after 6 weeks

*Statistically significant at *p* ≤ 0.05

Analysis confirmed that PAC-QoL scores improved significantly in both groups. In group A, the mean PAC-QoL score decreased from 51.60 ± 5.70 (median 51.0; range 42.0–62.0) at baseline to 18.12 ± 15.88 (median 11.0; range 7.0–55.0) at 6 weeks (*p* < 0.001, Wilcoxon test). In group B, scores also improved from 51.80 ± 5.67 (median 51.0; range 44.0–61.0) to 32.32 ± 15.34 (median 25.0; range 14.0–58.0) (*p* < 0.001). Between-group comparison showed a significantly greater reduction in PAC-QoL in group A compared to group B (*p* < 0.001, Mann–Whitney test) (Table 4, Fig. 5).

**Table 4:** Comparison of Patient Assessment of Constipation–Quality of Life (PAC-QoL) scores before and after 6 weeks of treatment

PAC-QoL	Group A (*n* = 25)	Group B (*n* = 25)	*U*	*p*
Pre-treatment				
Min–Max	42.0–62.0	44.0–61.0	307.0	0.915
Mean ± SD	51.60 ± 5.70	51.80 ± 5.67		
Median	51.0	51.0		
After 6 weeks				
Min–Max	7.0–55.0	14.0–58.0	100.0*	< 0.001*
Mean ± SD	18.12 ± 15.88	32.32 ± 15.34		
Median	11.0	25.0		
P1	< 0.001*	< 0.001*		

Both groups showed significant improvement, with a more pronounced reduction in group A. Data are presented as mean ± SD. Between-group differences were statistically significant (*p* < 0.001). Group A: Bilateral posterior tibial nerve stimulation. Group B: Control taking only medical treatment

*U* Mann–Whitney test,* p* *p* value for comparison between the two groups,* P1 p* value for Wilcoxon signed ranks test for comparison between pre-treatment and after 6 weeks

*Statistically significant at *p* ≤ 0.05

### Sex-based subgroup analysis

Sex-based subgroup analysis revealed differential treatment responses (Table 2). Among group A patients, women demonstrated significantly greater MODS improvement at 6 weeks (4.53 ± 0.72) compared to men (12.38 ± 6.21; *p* < 0.001). A similar trend was observed in group B (women 10.20 ± 1.47 vs. men 13.60 ± 1.51; *p* < 0.001). While baseline MODS scores were comparable between sexes in group A (*p* = 0.180), men in group B had significantly lower scores than women at baseline (*p* = 0.012) (Table 2).

Regarding PAC-QoL, baseline values were similar across sexes in both groups. However, after treatment, group A women showed a significantly greater reduction in PAC-QoL (10.76 ± 2.36) than men (33.75 ± 21.00; *p* < 0.001), and the same pattern was seen in group B (women 20.67 ± 4.10; men 49.80 ± 5.96; *p* < 0.001). Between-group comparisons confirmed that the improvement in PAC-QoL was significantly greater in women receiving BT-PTNS compared to those receiving medical treatment alone (*p* < 0.001). In contrast, in men, the difference was less pronounced but still statistically significant (*p* = 0.034) (Table 2).

### Electromyography outcomes

**Fig. 6:**
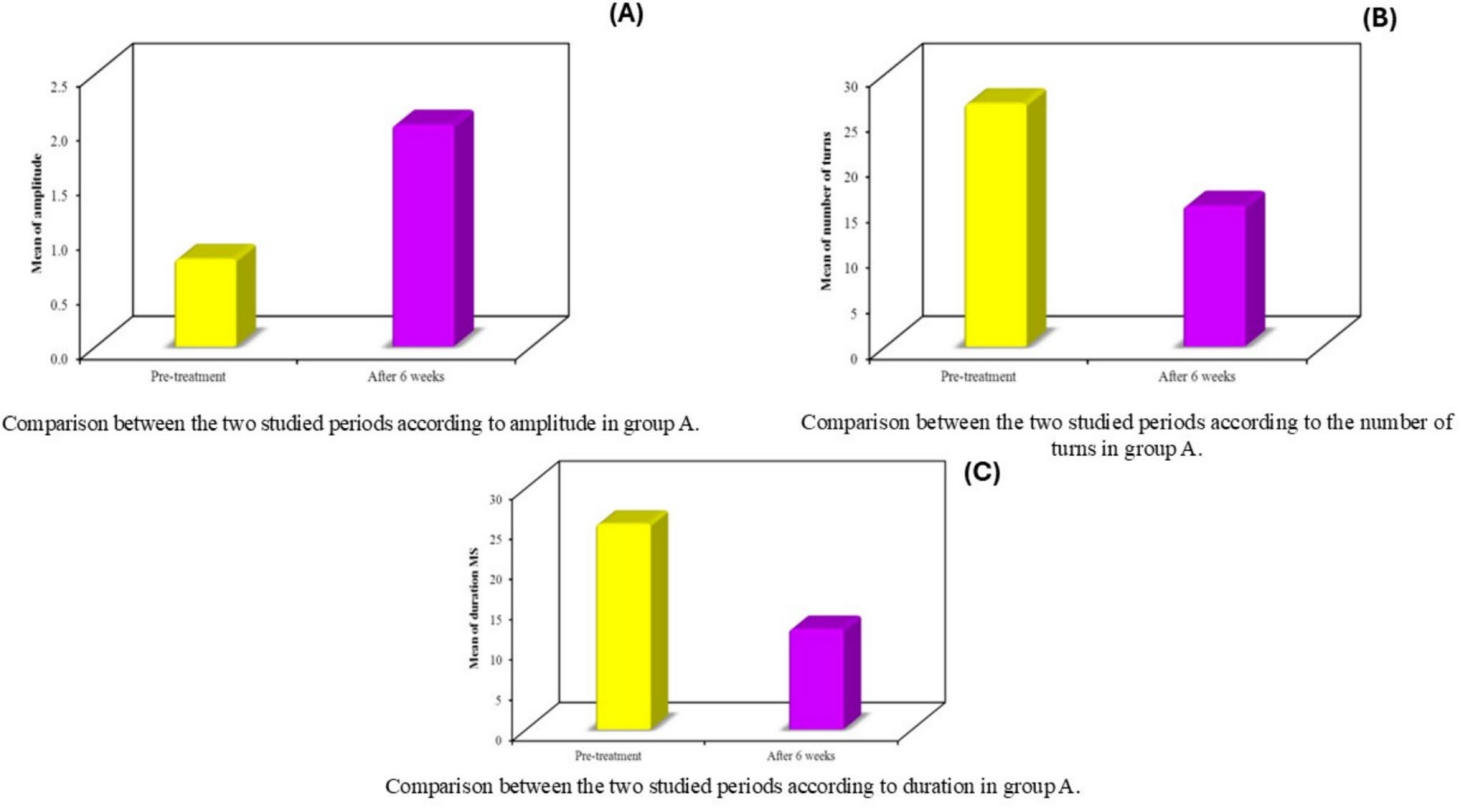
Electromyography (EMG) changes in group A before and after BT-PTNS treatment. **a** Amplitude (mV); **b** number of motor unit turns; **c** duration (ms). All EMG parameters showed statistically significant improvement after treatment (*p* < 0.001)

Quantitative EMG analysis in group A demonstrated significant improvements in all pelvic floor neuromuscular parameters after 6 weeks of BT-PTNS. EMG amplitude increased from a median of 0.80 mV (range 0.60–1.10) to 2.00 mV (range 1.60–2.50) (*p* < 0.001) (Table 5, Fig. 6). The number of motor unit turns decreased from a median of 27 (range 22–31) to 16 (range 12–18) (*p* < 0.001) (Table 6, Fig. 6), while motor unit duration decreased from a median of 26 ms (range 22–29) to 12 ms (range 9–16) (*p* < 0.001) (Table 7, Fig. 6).

**Table 5:** Comparison between the two studied periods according to amplitude in group A

	Pre-treatment (*n* = 25)	After 6 weeks (*n* = 25)	*t*^a^	*p*^b^
Amplitude				
Min–Max	0.60–1.10	1.60–2.50	20.240*	< 0.001*
Mean ± SD	0.80 ± 0.13	2.03 ± 0.24		
Median	0.80	2.0		

^a^*t* value for paired* t* test for comparison between preoperative and 6 months

^b^*p* value for paired *t* test for comparison between preoperative and 6 months

*Statistically significant at *p* ≤ 0.05

**Table 6:** Comparison between the two studied periods according to number of motor unit turns in group A

	Pre-treatment (*n* = 25)	After 6 weeks (*n* = 25)	*t*^a^	*p*^b^
Number of turns				
Min–Max	22.0–31.0	12.0–18.0	18.755*	< 0.001*
Mean ± SD	26.72 ± 2.26	15.44 ± 1.83		
Median	27.0	16.0		

^a^*t* value for paired* t* test for comparison between preoperative and 6 months

^b^*p* value for paired *t* test for comparison between preoperative and 6 months

*Statistically significant at *p* ≤ 0.05

**Table 7:** Comparison between the two studied periods according to duration in group A

	Pre-treatment (*n* = 25)	After 6 weeks (*n* = 25)	*t*^a^	*p*^b^
Duration MS				
Min–Max	22.0–29.0	9.0–16.0	24.585*	< 0.001*
Mean ± SD	25.52 ± 1.94	12.44 ± 1.85		
Median	26.0	12.0		

^a^*t* value for paired* t* test for comparison between preoperative and 6 months

^b^*p* value for paired *t* test for comparison between preoperative and 6 months

*Statistically significant at *p* ≤ 0.05

Sex-stratified EMG analysis revealed that all women in group A (100%) showed post-treatment improvement in EMG parameters, compared to only 3 of 8 men (37.5%) (*p* = 0.001), reinforcing the observed sex difference in clinical outcomes (Table 2).

No adverse events or complications were reported in either group during the study period.

## Discussion

Obstructed defecation is one of the pelvic floor disorders, which is a common source of morbidity in the developed world [1]. There are different modalities for the management of non-anatomic causes of obstructed defecation. One of them is the posterior tibial nerve stimulation, which we used in our study.

Since different outcome measures and inclusion criteria have been used, a comparison with the results of other studies is complicated. Currently, there is no standard criterion that exists as to which scoring system should be used.

In the Ieva Stundiene et al. study, 49 patients with constipation resistant to maximal conservative therapy were treated with PTNS twice a week for 6 weeks [26–30]. If the treatment was successful, patients were asked to continue the treatment for 6 months.

The effect was seen in 53.1% of patients. The mean Knowles–Eccersley–Scott Symptom Score improved significantly (from 20.88 ± 5.19 to 15.61 ± 7.19, *p* < 0.001) after 6 weeks. The 2-week stool frequency increased from the mean of 4.65 ± 2.48 pre-treatment to 7.47 ± 3.51 post-treatment (*p* < 0.001). The use of laxatives decreased (*p* < 0.001). The Gastrointestinal Quality of Life index improved in all subscales (*p* < 0.05).

The therapy was well tolerated, and no participant experienced any adverse event. No potential predictors of treatment success were found [31–35].

In the Nina Zhang et al. study, 12 patients with chronic constipation were treated with transcutaneous neuromodulation (TN) for 1 h twice daily for 2weeks [29, 36–41]. Bowel habit diary, Patient Assessment of Constipation Symptom (PAC-SYM), and Patient Assessment of Constipation Quality of Life (PAC-QOL) were assessed. After 2 weeks of TN therapy, 83% patients had more than three bowel movements per week (*P* = 0.01). TN improved PAC-SYM and PAC-QOL scores (both *P* < 0.001). The PAC-QOL total score decreased by 64% compared to the baseline. It was concluded that needleless TN at the posterior tibial nerve is effective in chronic constipation.

In the Collins et al. study, 18 patients with chronic constipation had 12 sessions of 30 min of percutaneous tibial nerve stimulation [28]. The PAC-QOL showed significant improvement (median 2.31 to 1.43) with a decrease of 40%. Stool frequency increased (*P* = 0.048) and the use of laxatives decreased (*P* = 0.025). There was no change in colonic transit time (*P* = 0.45).

In the Madbouly et al. study, 36 patients with rectal evacuation disorder were treated with BT-PTNS for 30 min, three times weekly for six consecutive weeks [25]. Symptomatic successful outcome was reported in 17 patients (47%), and the MODS decreased over 6 weeks (mean decrease = 10 points). Patients with a successful outcome (responders) had relatively lower preoperative MODS compared with patients with an unsuccessful outcome (nonresponders).

In the successful group, there was significant improvement after 6 weeks in patient assessment of Constipation–Quality of life score (mean improvement = 43.0 points). No significant change in Patient assessment of Constipation–Quality of life or rectal sensitivity was observed in the nonresponders.

In this study, 50 patients with obstructed defecation were included. Twenty-five patients received BT-PTNS (group A), and the other 25 patients received only medical treatment (group B). Group A was treated with BT-PTNS for a 30-min duration each, three times weekly over a period ranging from 6 to 12 weeks according to the patient's improvement. Group B took medical treatment in the form of dietary modification, including fiber and fluid supplementation and osmotic laxatives such as polyethylene glycol and lactulose for the same period as group A.

As regards the MODS score, there were statistically significant differences for both groups and between the two groups in comparison between pre-treatment and after 6 weeks. MODS in group A decreased over 6 weeks (mean decrease = 10 points) more than in group B (mean decrease = 4 points). The decrease in group A over 6 weeks (mean decrease = 10 points) was the same as in the Madbouly et al. study [25].

Also, there was significant improvement after 6 weeks in Patient assessment of Constipation–Quality of life score (mean improvement = 33 points) in group A, which is lower by 10 points than that from Madbouly et al.’s study [25]. While in group B, the mean improvement in Patient assessment of Constipation–Quality of life score was 19 points.

In this study, the PAC-QOL total score in group A was decreased by 65%. In the Nina Zhang et al. [43] study, Collins et al. study, and in the Madbouly et al. study, the PAC-QOL total score was decreased by 64%, 40% and 80%, respectively [25, 28].

Also, in this study, there was a statistically significant difference between pre-treatment and after 6 weeks as regards the quantitative EMG on pelvic floor muscles in group A in the amplitude, number of motor unit turns, and duration.

While our study was designed to assess short-term outcomes at 6 weeks, we acknowledge that the durability of treatment response remains an important question. Previous studies, such as those by Stundienė et al. [42], have reported symptom improvement sustained for up to 6 months with continued PTNS. However, long-term effectiveness and the potential need for maintenance stimulation protocols remain uncertain. Future studies with extended follow-up are warranted to evaluate the lasting impact of BT-PTNS and to determine optimal treatment frequency and duration.

This study represents one of the few randomized controlled trials investigating the efficacy of BT-PTNS in patients with functional ODS. A key strength lies in the use of both validated symptom scores (MODS and PAC-QoL) and objective neurophysiological assessments via quantitative EMG, allowing for a multidimensional evaluation of treatment effects. Additionally, the inclusion of a well-matched control group receiving standard medical therapy enhances the internal validity of the findings. The study also offers novel sex-stratified subgroup analysis, revealing clinically relevant differences in treatment response between male and female participants.

However, this study has several limitations. The sample size was relatively small, and the follow-up period was limited to 6 weeks, which precludes conclusions regarding the long-term durability of BT-PTNS effects. While short-term improvements were robust, future studies with extended follow-up are necessary to assess whether benefits are maintained beyond the treatment period or require maintenance stimulation protocols. An important limitation of our study is the imbalance in symptom duration between sexes. Most male participants reported symptoms lasting ≥ 5 years, whereas the majority of female participants had symptoms of < 5 years. This discrepancy may partly explain the poorer outcomes observed in men, not necessarily due to sex-specific biological differences, but rather due to the negative impact of chronicity on neuromuscular adaptability and treatment responsiveness. Longer disease duration may be associated with more entrenched pelvic floor dysfunction or irreversible changes, thereby attenuating the therapeutic effect of neuromodulation. At the same time, we cannot fully exclude the possibility of true sex-related differences in treatment response. Although our study was adequately powered to assess overall treatment effects, it was not designed to stratify outcomes by both sex and symptom duration simultaneously; thus, we could not perform multivariable modeling to formally test these hypotheses. However, the observed distribution in our cohort strongly suggests that symptom duration may confound the apparent sex effect. Future studies with larger, stratified cohorts should adjust for symptom duration at baseline while also exploring potential biological sex-related factors.

## Conclusion

Bilateral transcutaneous posterior tibial nerve stimulation (BT-PTNS) is a safe and effective noninvasive intervention for improving symptoms and quality of life in patients with functional ODS. When added to standard medical therapy, BT-PTNS resulted in significantly greater improvements in both subjective and objective outcomes compared to medical therapy alone. Female participants demonstrated a more favorable neuromuscular and clinical response, highlighting the importance of sex-based considerations in future research.

## Data Availability

The datasets generated and/or analyzed during the current study are not publicly available due to concerns regarding patient privacy and confidentiality, per ethical and institutional guidelines. Still, they are available from the corresponding author on reasonable request.
